# Magnetic Beads-Based Sensor with Tailored Sensitivity for Rapid and Single-Step Amperometric Determination of miRNAs

**DOI:** 10.3390/ijms18112151

**Published:** 2017-11-09

**Authors:** Eva Vargas, Rebeca M. Torrente-Rodríguez, Víctor Ruiz-Valdepeñas Montiel, Eloy Povedano, María Pedrero, Juan J. Montoya, Susana Campuzano, José M. Pingarrón

**Affiliations:** 1 Department of Analytical Chemistry, Faculty of Chemistry, University Complutense of Madrid, 28040 Madrid, Spain; evargas_orgaz@hotmail.com (E.V.); rebeca.magnolia@gmail.com (R.M.T.-R.); victor_lega90@hotmail.com (V.R.-V.M.); elpove01@ucm.es (E.P.); mpedrero@quim.ucm.es (M.P.); 2 Cannan Research and Investment & Faculty of Medicine, University Complutense of Madrid, 28040 Madrid, Spain; jjmontoya@canaanrd.com

**Keywords:** miRNA-21, electrochemical sensor, anti DNA–RNA hybrid antibody, ProtA-PolyHRP40, cancer cells, breast human tissues

## Abstract

This work describes a sensitive amperometric magneto-biosensor for single-step and rapid determination of microRNAs (miRNAs). The developed strategy involves the use of direct hybridization of the target miRNA (miRNA-21) with a specific biotinylated DNA probe immobilized on streptavidin-modified magnetic beads (MBs), and labeling of the resulting heteroduplexes with a specific DNA–RNA antibody and the bacterial protein A (ProtA) conjugated with an horseradish peroxidase (HRP) homopolymer (Poly-HRP40) as an enzymatic label for signal amplification. Amperometric detection is performed upon magnetic capture of the modified MBs onto the working electrode surface of disposable screen-printed carbon electrodes (SPCEs) using the H_2_O_2_/hydroquinone (HQ) system. The magnitude of the cathodic signal obtained at −0.20 V (vs. the Ag pseudo-reference electrode) demonstrated linear dependence with the concentration of the synthetic target miRNA over the 1.0 to 100 pM range. The method provided a detection limit (LOD) of 10 attomoles (in a 25 μL sample) without any target miRNA amplification in just 30 min (once the DNA capture probe-MBs were prepared). This approach shows improved sensitivity compared with that of biosensors constructed with the same anti-DNA–RNA Ab as capture instead of a detector antibody and further labeling with a Strep-HRP conjugate instead of the Poly-HRP40 homopolymer. The developed strategy involves a single step working protocol, as well as the possibility to tailor the sensitivity by enlarging the length of the DNA/miRNA heteroduplexes using additional probes and/or performing the labelling with ProtA conjugated with homopolymers prepared with different numbers of HRP molecules. The practical usefulness was demonstrated by determination of the endogenous levels of the mature target miRNA in 250 ng raw total RNA (RNA_t_) extracted from human mammary epithelial normal (MCF-10A) and cancer (MCF-7) cells and tumor tissues.

## 1. Introduction

Cancer is a leading global cause of death that needs appropriate diagnosis methods. It is well-known that tissue biopsies—an invasive test on which most therapy decisions are based—provide just a static snapshot of the tumor instead of the constantly evolving entire tumor genome. Therefore, it is essential to develop alternative diagnosis strategies able to detect the disease at early stages and monitor the real-time dynamic of cancer, preferably in a noninvasive way. Recent advances have highlighted the clinical potential of specific microRNAs (miRNAs) in early tumor diagnosis and clinical management, but also the challenges involved in their determination. miRNAs are short, non-coding single-stranded RNA molecules, approximately 22 nucleotides in length, acting at the post-transcriptional level by partially binding to the mRNA of genes that play key roles in the regulation of as much as 30% of all mammalian protein-encoding genes [1]. These relevant biomarkers have demonstrated involvement both in a wide range of biological processes (cell cycle control, apoptosis, and several developmental and physiological processes) in healthy individuals, and in a number of diseases such as cancer, heart and neurological diseases. Indeed, the expression of miRNAs has been demonstrated to be closely related to the regulation and progression of numerous cancers, and their expression levels can provide useful information on the diagnosis, classification, and treatment of cancer patients [2]. However, the determination of these clinically relevant biomarkers needs to overcome important difficulties such as their low abundance; short length, which makes traditional DNA-based methods not sensitive enough for a reliable detection; and sequence similarity in family members, which demands highly selective methodologies able to discriminate single nucleotide mismatches [2,3,4].

Current strategies for miRNA determination include northern blotting [5,6], in situ hybridization, reverse-transcription polymerase chain reaction (RT-PCR) [7,8,9], and miRNA microarrays [10,11,12]. Although these powerful technologies have led to significant contributions for miRNA determination, wide application is still hindered by expensiveness, low sensitivity, tedious steps, harmful reagents, and sophisticated and non-portable instruments restricting their use to central laboratories. Therefore, there is a pressing need to develop simple, sensitive, rapid, portable, and low-cost methodologies for miRNA determination. Accordingly, electrochemical biosensors have evolved dramatically over the past decades and have demonstrated very attractive capabilities for miRNAs determination [13,14,15,16,17,18,19,20,21,22,23]. However, most of the developed biosensors achieve enhanced sensitivities using complicated amplification strategies, and show a lack of simplicity in their fabrication process while demanding long times to perform the determination; these characteristics restrict their wider use in clinical applications. Particularly attractive and relatively simple strategies for the development of electrochemical biosensors for miRNA determination have been reported recently by our group, involving the use of viral proteins [24] or antibodies [25] as versatile bioreceptors specifically directed to RNA/RNA and RNA/DNA hybrids, respectively [26]. Taking advantage of the unique binding properties demonstrated by antibodies against RNA/DNA hybrids, we report here a new disposable electrochemical platform for sensitive and rapid miRNA determination based on direct hybridization of a specific DNA capture probe immobilized onto Streptavidin-functionalized magnetic microcarriers (Strep-MBs) with the target miRNA, recognition of the captured DNA–miRNA heteroduplexes with a suitable antibody, and labeling with a bacterial protein conjugated with an HRP homopolymer (ProtA-PolyHRP40). Therefore, the main novelty of this approach compared to the previous one [25] using the same anti-DNA–RNA hybrid antibody (apart from the differences in the functionalized MBs used as solid support) is that the antibody acts as detector bioreceptor, instead of as capture antibody, of the DNA–miRNA heteroduplexes previously attached to the Strep-MBs. Another important difference is the use of ProtA–PolyHRP40 for antibody labelling. As a result of this novel strategy, the resulting biosensor exhibits interesting practical advantages in terms of sensitivity, reduced number of steps involved in the working protocol, and lower assay time. The variation in the cathodic current measured using the H_2_O_2_/hydroquinone (HQ) system, after magnetic capture of the modified MBs onto screen-printed carbon electrodes (SPCEs), is related with the concentration of the target miRNA in the analyzed sample. The proposed biosensor exhibited excellent sensitivity (detection limit (LOD) of 0.4 pM) and feasibility to perform a 30 min accurate determination of the target miRNAs using a low mass amount of raw total RNA (RNA_t_) extracted from cancer cells and human breast tissues.

## 2. Results and Discussion

**Figure 1:**
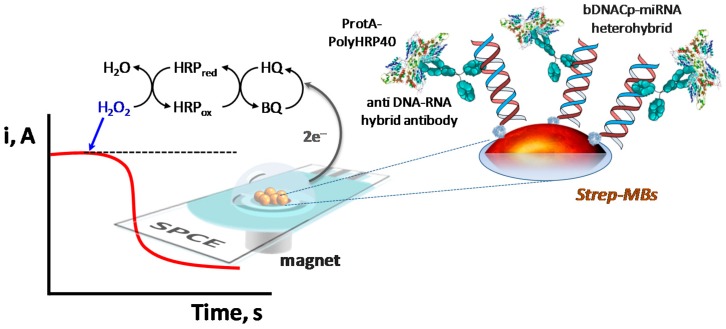
Schematic illustration of the fundamentals involved in the amperometric biosensor developed for the determination of microRNAs (miRNAs) based on the efficient formation of b-DNACp–miRNA heteroduplexes on the surface of Streptavidin-functionalized magnetic microcarriers (Strep-MBs) and their selective labeling with the anti-DNA–RNA hybrid antibody and ProtA–PolyHRP40. SPCE: screen-printed carbon electrode; b-DNACp: biotinylated complementary DNA probe.

The strategy for miRNA determination we report here relies on the efficient hybridization of the target miRNA with a biotinylated complementary DNA probe (b-DNACp) immobilized onto Strep-MBs; recognition of the perfectly matched DNA–miRNA heterohybrids with an antibody specific for DNA–RNA heteroduplexes, labeled with protein A conjugated with an HRP homopolymer (ProtA–PolyHRP40); and amperometric detection at SPCEs. This approach is schematized in Figure 1 and involves two main steps: (i) selective capture of the target miRNA at the b-DNACp-modified MBs and simultaneous labeling of the b-DNACp–miRNA heteroduplexes with the anti-DNA–RNA hybrid antibody and ProtA–PolyHRP40, and (ii) amperometric detection of the cathodic current produced upon addition of H_2_O_2_, using HQ as a redox mediator in solution, after the magnetic capture of the modified MBs on the working electrode surface of the SPCE. The measured magnitude of the cathodic current is related to the amount of HRP immobilized on the surface of the MBs, this being in turn related to the number of hetero-duplexes formed and, therefore, proportional to the concentration of the target miRNA in the sample.

As a model miRNA target, miRNA-21, considered as a promising biomarker and therapeutic target for cancer, was selected to evaluate the proposed method feasibility, optimize all the involved experimental variables, and evaluate the analytical performance of the methodology.

### 2.1. Optimization of the Experimental Variables

All the experimental variables involved in the preparation of the electrochemical biosensor for miRNAs were optimized by taking as the selection criterion the largest ratio between the current values measured at a potential value of −0.20 V (vs. the Ag pseudo-reference electrode), previously optimized for the HRP/HQ/H_2_O_2_ system [27], in the absence (N) and in the presence of 100 pM (S) miRNA-21. The evaluated variables, the tested ranges, and the values selected for further work are summarized in Table 1.

**Figure 2:**
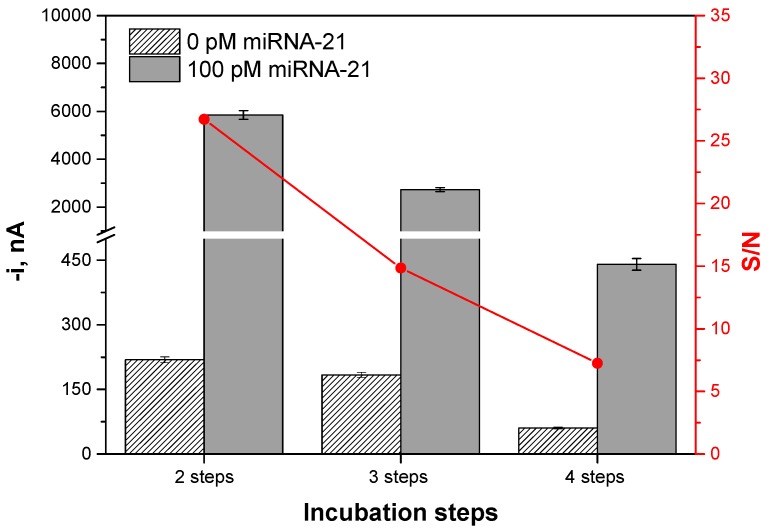
Comparison of the amperometric responses obtained in the absence (N) and in the presence of 100 pM (S) miRNA-21 and the resulting signal-to-noise (S/N) current ratios, when the biosensor was prepared using working protocols with a different number of incubation steps. Error bars estimated at triple of the standard deviation of three replicates.

The influence of the number of steps used in the preparation of the biosensor was also investigated in search of a protocol simplification to also reduce the assay time. All procedures, described in Table 2, involved 30 min incubation steps. Figure 2 shows a comparison between the resulting amperometric responses and the corresponding signal-to-noise (S/N) ratios for the protocols carried out. As it can be seen, the working protocol involving only two incubation steps provided the largest S/N current ratio which, additionally, allows a large reduction in the total assay time.

**Figure 3:**
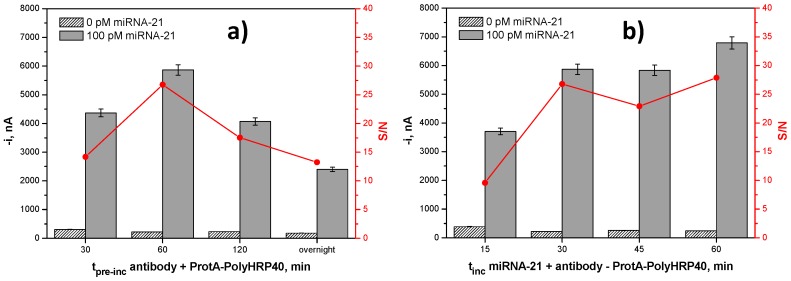
Dependence of the amperometric responses obtained with the developed biosensor in the absence (N) and in the presence of 100 pM (S) miRNA-21 and the resulting S/N current ratios, with the pre-incubation time of anti-DNA–RNA hybrid antibody and ProtA–PolyHRP40 mixture solution (**a**), and with the incubation time of b-DNACp-MBs with the mixture solution containing the target miRNA and the anti-DNA–RNA hybrid antibody preincubated for 1 h with the ProtA–PolyHRP40 (**b**). Error bars estimated at triple of the standard deviation of three replicates.

From these results, it was deduced that a better recognition of the anti-DNA–RNA hybrid antibody by ProtA–PolyHRP40 occurred when both reagents were free in solution. Therefore, the effect of the pre-incubation time of the solution containing these reagents on the recognition efficiency of the anti-DNA–RNA hybrid antibody Fc region by the ProtA–PolyHRP40 was evaluated (Figure 3a). The S/N ratio increased with the incubation time up to 60 min and then decreased significantly, probably due to hindered recognition by steric or agglutination effects. In addition, the effect of the incubation time of the b-DNACp-MBs with the mixture solution containing the target miRNA and the anti-DNA–RNA hybrid antibody preincubated for 1 h with the ProtA–PolyHRP40 was tested (Figure 3b). Similar large S/N current ratios were observed for 30 and 60 min, 30 min being then considered as an incubation time sufficient to ensure high efficiency in the hybridization and labeling events.

### 2.2. Analytical Characteristics

The reproducibility of the amperometric responses obtained with different biosensors prepared in the same manner was evaluated by comparing the current values measured for 25 pM of miRNA-21. The measurements made with ten different sensors provided a relative standard deviation (RSD) value of 3.1%, which demonstrated the great reproducibility of both the sensor fabrication and the amperometric transduction protocols used.

**Figure 4:**
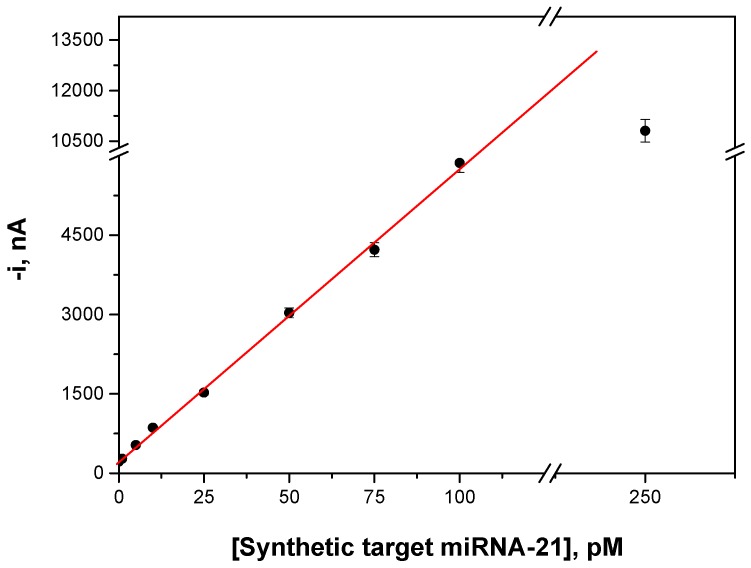
Calibration plot constructed for miRNA-21 with the developed electrochemical biosensor. Error bars estimated at triple the standard deviation of three replicates.

Figure 4 displays the calibration curve constructed for the synthetic target miRNA and Table 3 summarizes the corresponding analytical characteristics, showing an estimated LOD as low as 0.4 pM (10 attomoles in a 25 μL sample).

The storage stability of the b-DNACp-MBs was evaluated by keeping them at 4 °C in microcentrifuge tubes containing 50 μL of filtered phosphate-buffered saline (PBS). Each working day, the amperometric responses obtained with sensors prepared using the stored b-DNACp-MBs for 0.0 and 25 pM miRNA-21 solutions were measured. No significant decrease in the resultant S/N ratio was observed during 17 days, suggesting the possibility of preparing b-DNACp-MBs in advance and storing them under the above-described conditions until the biosensor preparation is required.

The analytical performance of this method was compared with that reported for other electrochemical biosensors involving different amplification strategies [21,22,23]. As expected, a considerably higher LOD was achieved with this rapid and single-step method (0.4 pM) vs. the low femtomolar level attained with the methods using amplification strategies. However, it is important to emphasize that the method reported here offers important practical advantages such as a considerable shortening of the assay time and a much simpler working protocol. The mentioned amplification-using methodologies require long procedures to modify the electrode surface [21,23], application of a high hybridization temperature [21], or protocols lasting more than 24 h for the preparation of nanomaterial bioconjugates for amplification purposes [22]. Moreover, as it is shown below, the sensitivity achieved with the methodology presented in this work is sufficient to allow the determination of the target miRNA in breast tumor cells and tissues.

In comparison with a previously described electrochemical biosensor for miRNA-21 using anti-DNA–RNA hybrid antibodies as capture antibodies and further labeling with a Strep-HRP conjugate [25], a six-times-lower LOD (0.4 vs. 2.4 pM) and a six-times-higher sensitivity (55,314 vs. 9548 nA nM^−1^) were achieved using the strategy reported in this work. These improvements can be attributed both to the use of the anti-DNA–RNA hybrid antibody as detector instead of capture bioreceptor and to the small size of its binding epitope. As shown by Qavi et al. [28], the binding epitope is of the order of six base pairs in size and approximately three anti-DNA–RNA hybrid antibodies can bind per bDNACp–miRNA duplex. Furthermore, results also demonstrate that the use of ProtA conjugated with HRP homopolymers is an interesting strategy for signal amplification. In fact, a 120-times-enhanced sensitivity was achieved by using ProtA–HRP40 instead of the conventional ProtA–HRP (slope values of 55,314 vs. 459 nA nM^−1^) for getting the electrochemical signal. It is worthwhile to note that the LOD achieved in this work is also remarkably better than those reported for other non-electrochemical methodologies. For instance, the LOD of the amperometric magneto-biosensor is more than 3000 times lower than that achieved in a recent label-free method for miRNA-222 determination using a two-step hybridization assay with Surface Enhanced Raman Scattering (SERS)-based microfluidic polydimethylsiloxane (PDMS) chips integrating silver-coated porous silicon membranes [29] (0.4 pM vs. 1.51 nM).

Apart from the sensitivity, additional advantages compared to the previously reported methodology [25] include a simpler working methodology by reducing both the steps involved in the protocol from two to one (once the bioreceptor-modified MBs are prepared and the anti-DNA–RNA hybrid antibody and ProtA–PolyHRP40 have been preincubated for 1 h), and the assay time from 75 to 30 min. Interestingly, the great enhancement in sensitivity demonstrated by using the anti-DNA–RNA hybrid antibody for detection and ProtA–PolyHRP40 for labeling suggests the possibility of tailoring the sensitivity of the approach for a particular application (concentration level of the target miRNA to be detected) by enlarging the length of the DNA–miRNA heteroduplexes using additional probes leading to higher numbers of antibodies attached to the heterohybrids and/or performing the labelling with ProtA conjugated with different numbers of HRP-containing homopolymers (commercially available with 20, 40, and 80 HRP molecules).

Moreover, in the cases where the test time is decisive, the loss of sensitivity provoked by shortening the incubation time of the b-DNACp-MBs with the target miRNA, anti-DNA–RNA hybrid antibody, and ProtA–HRP40 mixture solution down to 15 min (shown in Figure 3b), can be counteracted by employing a larger concentration of the labeling bioreagents. Data presented in Table 4 show how the 37% loss in sensitivity observed by halving the optimal incubation time turns into only a 2.4% loss just by doubling the ProtA–HRP40 concentration. These relevant results outline the potentiality of the developed methodology to be employed as a 15 min method for the sensitive and straightforward determination of miRNAs.

### 2.3. Selectivity

**Figure 5:**
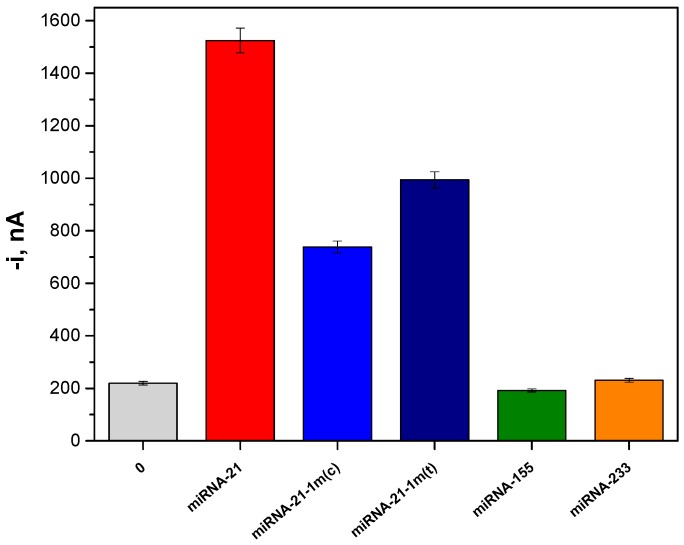
Selectivity of the developed electrochemical biosensor for determination of miRNA-21. Amperometric responses measured in the absence or in the presence of 25 pM miRNA-21, central (1-m(c)), terminal (1-m(t)), and non-complementary (NC) sequences (miRNA-155 and miRNA-223). Error bars estimated at triple the standard deviation of three replicates.

The feasibility of the developed biosensor to selectively determine the target miRNA in complex mixtures also containing many other miRNAs and oligonucleotides was demonstrated by comparing the amperometric signals obtained in the absence and in the presence of 25 pM miRNA-21, as well as for two single-base mismatched sequences in the central (1-m(c)) or terminal (1-m(t)) position and two fully non-complementary (NC) sequences (miRNA-155 and miRNA-223). As shown in Figure 5, the amperometric responses obtained with the NC sequences were similar to those measured in the absence of target miRNA, while the 1-m(c) and 1-m(t) sequences gave 48% and 65% of the response provided by the target miRNA, respectively. This discrimination towards the 1-m miRNAs, even in non-stringent hybridization conditions, were of the same order (51% for a 1-m(c)) to that reported previously for the electrochemical biosensor constructed with the same anti-DNA–RNA Ab as capture antibody [25], and much better than that reported (83%) using p19 modified-MBs-based amperometric biosensors [24].

### 2.4. Determination of Mature miRNA-21 in RNA_t_ Extracted from Cancer Cells and Tumor Tissues

The developed methodology was applied to the determination of the endogenous content of mature miRNA-21 in raw RNA_t_ extracted from breast cancer (MCF-7) vs. nontumorigenic epithelial (MCF-10A) cells and human tumor (T) vs. paired normal adjacent (NT) breast tissues.

**Figure 6:**
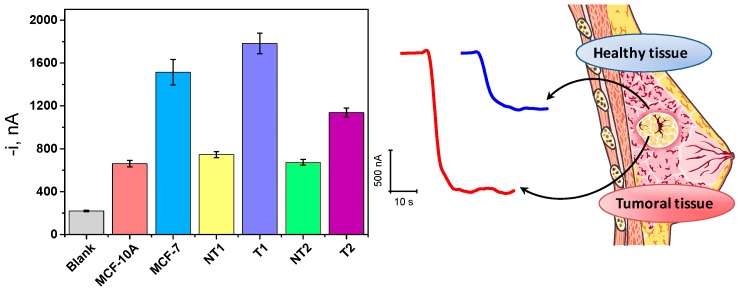
Amperometric responses measured with the developed biosensor for the determination of the endogenous content of miRNA-21 in 250 ng raw total RNA (RNA_t_) extracted from cells and human breast tissues. Amperograms obtained for human tumor (T) and paired normal adjacent (NT) samples extracted from a breast cancer patient are also shown. Error bars estimated at triple the standard deviation of three replicates.

The amperometric responses obtained for the analysis of 250 ng of RNA_t_ extracted from these samples are shown in Figure 6 and indicate overexpression of miRNA-21 in cancer cells and breast tumor tissues compared with normal cells and healthy tissues, which is in agreement with previous reports [24,25,30,31,32] and with the oncogenic function of miRNA-21 [33] in breast cancer.

The possible existence of a matrix effect for quantification in these samples was tested by constructing a calibration plot prepared by spiking 250 ng of extracted RNA_t_ samples with growing concentrations of synthetic miRNA-21 up to 10 pM. The slope value of the linear calibration plot was significantly lower, approximately 10%, than that calculated from the calibration graph constructed with the synthetic target miRNA-21 in the buffered solutions. Therefore, the existence of a matrix effect was concluded, possibly due to a hindered efficiency of the b-DNACp–miRNA-21 heterohybrids recognition by the antibody and ProtA–PolyHRP in the RNA_t_ samples. Accordingly, the endogenous concentration of the mature target miRNA in all these samples was determined by applying the standard additions method. The results obtained are summarized in Table 5. It is important to mention here that, apart from the matrix effect, a significant decrease in the amperometric responses were observed when RNA_t_ amounts larger than 250 ng were used. This was attributed to a hook effect occurring when the amount of the endogenous target miRNA exceeds, in a large amount that of the bDNACp immobilized on the MBs [34]. It is important to note also that no significant matrix effect was observed when the hybridization and labelling steps were performed sequentially. Indeed, slope values of (20.3 ± 0.7), (20 ± 3), and (21 ± 2) nA pM^−1^ were obtained for synthetic microRNA and in the presence of 250 ng of RNA_t_ extracted from MCF-10A cells and T1, respectively. These results indicate that the matrix effect was due to the worse efficiency of the labelling step in the presence of RNA_t_. Nevertheless, despite the existence of the matrix effect, we think that the one-step protocol can be considered as more advisable to perform the miRNA determination because of its rapidity, straightforwardness, and higher sensitivity (see Figure 2 to compare the achieved sensitivities when the hybridization and labeling steps are carried out simultaneously or sequentially).

It is important to remark that data in Table 5 show that the miRNA-21 concentrations found in cells agree with those reported previously, as well as within the ranges in breast tissues reported by other authors, thus confirming the suitability of the developed method for the accurate and selective determination of target miRNAs in complex samples where other nontarget miRNAs are also present in a large extent. It is worthwhile to also remark that the sample mass amount used in this work is between two and four times lower than that required with other electrochemical sensors [24,25].

## 3. Materials and Methods

### 3.1. Apparatus and Electrodes

Amperometric measurements were performed with a CH Instruments (Austin, TX, USA) model 812B potentiostat controlled by software CHI812B. Screen-printed carbon electrodes (SPCEs) (DRP-110, DropSens, Asturias, Spain), consisting of a 4 mm diameter carbon working electrode, a carbon counter electrode, and an Ag pseudo-reference electrode were used as electrochemical transducers. A specific cable connector (DRP-CAC, DropSens, S.L.) acted as interface between the SPCEs and the potentiostat. All measurements were performed at room temperature. A neodymium magnet (AIMAN GZ, Madrid, Spain) embedded in a homemade Teflon casing was used to magnetically capture, in a reproducible way, the modified-MBs on the surface of the SPCEs.

A Raypa steam sterilizer, a Telstar Biostar biological safety cabinet, an Optic Ivymen^®^ System incubator shaker (Comecta S.A, Sharlab, Madrid, Spain), a Bunsen AGT-9 Vortex for homogenization of the solutions, a DynaMag™-2 magnetic particle concentrator (123.21D, Invitrogen Dynal AS, Oslo, Norway), and a Sigma-Aldrich 1–15 K refrigerated microcentrifuge were also employed. The quality and quantity of the extracted RNA were evaluated by using a NanoDrop^®^ ND-1000 spectrophotometer (NanoDrop Technologies, Wilmington, DE, USA).

### 3.2. Reagents and Solutions

Highest analytical grade reagents were used in all cases. Streptavidin-modified magnetic beads (Strep-MBs, 2.8 μm·Ø, 10 mg·mL^−1^, Dynabeads M-280 Streptavidin, 11206D) were purchased from Dynal Biotech ASA (Oslo, Norway).

The reagents NaCl, KCl, NaH_2_PO_4_, Na_2_HPO_4_, and Tris-HCl were purchased from Scharlab (Barcelona, Spain). ProtA HRP conjugate (ProtA–HRP), hydroquinone (HQ), and H_2_O_2_ (30%, *w*/*v*) were purchased from Sigma-Aldrich (St. Louis, MO, USA), and ethylenediaminetetraacetic acid (EDTA) from Merck (‎Darmstadt, Germany). ProtA–PolyHRP40, a native ProtA labeled with a PolyHRP40, a homopolymer containing 40 HRP molecules covalently linked together (Catalog No.: ABIN929490, from antibodies-online) and an anti-DNA–RNA Hybrid (S9.6) antibody (AbS9.6) from Kerafast (Boston, MA, USA) were also purchased. A commercial blocker casein solution, consisting of a ready-to-use, PBS solution containing 1% *w*/*v* purified casein, was purchased from Thermo Scientific (Waltham, MA, USA). All the DNA and RNA synthetic oligonucleotides used, whose sequences are described in Table 6, were purchased from Sigma-Aldrich. Upon reception, they were reconstituted in nuclease-free water to a final concentration of 100 μM, divided into small aliquots, and stored at −80 °C.

To perform the amperometric detection, the modified-MBs were washed twice with 50 μL of blocker casein solution and re-suspended in 45 μL of 0.05 M sodium phosphate buffer solution (pH 6.0). All MB manipulations carried out before the amperometric measurements were performed in a laminar flow cabinet to avoid RNAse contamination and prevent miRNA degradation.

All the required buffer solutions were prepared in Milli-Q deionized water (18.2 MΩ cm): PBS, consisting of 0.01 M phosphate buffer solution supplemented with 0.137 M NaCl and 0.0027 M KCl, with pH 7.5; 0.05 M phosphate buffer, with pH 6.0; Binding and Washing buffer (B&W), consisting of 10 mM Tris-HCl solution containing 1 mM EDTA and 2 M NaCl, with pH 7.5 (sterilized after their preparation to avoid RNAses degradation); and 0.05 M phosphate buffer with pH 6.0.

### 3.3. MBs Modification

A quantity of 5.0 μL of the commercial Strep-MBs suspension was transferred into a microcentrifuge tube and washed twice with 50 μL B&W by placing the particles in the magnetic concentrator and discarding the supernatant after 3 min. After washing, MBs were incubated with 25 μL of 0.1 μM of the biotinylated antiDNA-21 capture probe solution (prepared in B&W) for 30 min (30 °C, 950 rpm). After two washing steps with 50 μL of blocker casein solution, b-antiDNA-21Cp-coated MBs were incubated for a duration of 30 min (950 rpm, 30 °C) in 25 μL of a solution prepared by mixing the synthetic target or the RNA_t_ extracted solution with a solution containing anti-DNA–RNA and ProtA–HRP40 (both bioreagents at 2 μg·mL^−1^) which was prepared in blocker casein solution and incubated previously for 1 h at room temperature. Simultaneous control experiments were performed in the absence of target miRNA to evaluate the blank signal.

### 3.4. Electrochemical Measurements

Amperometric measurements were made by allocating the SPCE on a homemade casing of Teflon with an encapsulated neodymium magnet. In this way the MBs were magnetically captured on the working carbon electrode in a reproducible and stable way by pipetting 45 μL of the modified MB suspension onto the SPCE. Then, the ensemble SPCE/magnet holding block was immersed into an electrochemical cell containing 10 mL of 0.05 M phosphate buffer of pH 6.0 and 1.0 mM HQ (prepared just before performing the electrochemical measurement). Amperometric measurements in stirred solutions were made at −0.20 V vs. an Ag pseudo-reference electrode. Once the baseline was stabilized, 50 μL of a 0.1 M H_2_O_2_ solution were added and the current recorded over approximately 100 s, until such time as the steady-state current was reached. The amperometric signals given through the manuscript, corresponding to the difference between the steady-state and the background currents, are the average of at least three replicates, being the confidence intervals calculated for α = 0.05.

### 3.5. Cell Culture, Human Tissues and RNA_t_ Extraction

Protocols used for cell culture, collection of human tissues, and RNA_t_ extraction were the same as described previously [25,36].

Briefly, breast cancer cells MCF-7 were grown at 37 °C in a humidified atmosphere containing 5% CO_2_ and maintained in high-glucose DMEM (Dulbecco’s modified Eagle’s medium), supplemented with fetal bovine serum (10%), penicillin (100 U mL^−1^), streptomycin (100 μg mL^−1^), and l-glutamine (2.5 mM) (GIBCO-Invitrogen, Carlsbad, CA, USA), whereas nontumorigenic epithelial MCF-10A cells were cultured in high-glucose DMEM/Ham’s Nutrient Mixture F12 (1:1) with l-glutamine (2.5 mM), horse serum (5%, Gibco), human insulin (10 mg·mL^−1^, Sigma-Aldrich), hydrocortisone (0.5 mg·mL^−1^, Sigma-Aldrich), Epidermal growth factor (EGF, 10 ng·mL^−1^), and cholera toxin (100 ng·mL^−1^, QuadraTech Diagnostics Ltd., Epsom, Surrey, UK).

With informed consent approved by Getafe University Hospital (Madrid, Spain) a 0.3 to 0.5 cm breast cancer tissue (T) and paired normal adjacent tissue (NT) from breast cancer patients were sectioned by the pathologist immediately after surgical excision of the tumor and placed in vials filled with RNAlater^®^ tissue storage reagent. This stabilization reagent was removed after 48 h of storage at 4 °C and the tissues kept frozen at −80 °C until their use.

RNA_t_ was isolated from all these samples using Tri Reagent (Molecular Research Center, Inc., Cincinnati, OH, USA). Briefly, PBS-washed cells were scraped off and spun down. After homogenizing the pellet for 5 min in Tri Reagent at room temperature, the extraction with chloroform was carried out and the RNA_t_, in the upper aqueous phase, was precipitated with isopropyl alcohol and washed twice in 70% EtOH. Finally, the pellet was dried out for a duration of 10 min in a heating plate at 80 °C, dissolved in RNase-free water, and stored at −80 °C [36]. The RNA_t_ quality and concentration were evaluated by measuring the absorbance at the appropriate wavelengths (260, 230, and 280 nm) with an ND-1000 spectrophotometer, obtaining ratio values confirming pure RNA in all cases.

## 4. Conclusions

This work describes a novel amperometric disposable biosensor for the rapid, facile, and sensitive detection of miRNAs. The method, illustrated for miRNA-21 determination, is based on the selective capture of the target miRNA by specific DNA capture probe-modified MBs, recognition of the resulting DNA–miRNA heteroduplexes by a specific antibody further labeled with a bacterial protein conjugated with a homopolymer containing multiple HRP molecules, and amperometric detection upon magnetic capture of the modified MBs onto a SPCE. The biosensor exhibits very interesting analytical performance using a simple approach, providing a LOD of 0.4 pM within 30 min, without requiring previous reverse transcription of RNA to cDNA, complex amplification protocols, or the use of an internal reference. In addition, the biosensor shows successful applicability in the analysis of raw RNA_t_ samples extracted from cancer cells and human tumor specimens. The short assay time, simplicity, and feasibility to tailor the final sensitivity, to be applied for the determination of any target RNA and to perform multiple analyses in a single experiment, position this versatile methodology as a promising tool for high-throughput and simple miRNAs/RNAs bioanalysis applicable to a broad range of settings.

## Figures and Tables

**Table 1:** Optimization of the experimental variables affecting the performance of the amperometric biosensor developed for miRNA-21 determination.

Experimental Variable	Tested Range	Selected Value
[b-DNACp], μM	0.0–1.0	0.1
t_b-DNACp incubation_, min	0–60	30
Strep-MBs, μL	2.5–10.0	5.0
Number of incubation steps	2–4	2
Anti-DNA-RNA hybrid antibody dilution	1:100–1:10,000	1:1000
ProtA–PolyHRP40 dilution	1:5–1:250	1:25
t_ProtA–HRP40 + AbS9.6 pre-incubation_, min	30–overnight	60
t_mixture solution incubation_, min	15–60	30

**Table 2:** Working protocols evaluated to prepare the amperometric biosensor for the determination of miRNA-21.

Incubation Steps (30 min Each)	Protocol	Total Assay Time, min
2	(1) b-DNACp immobilization onto Strep-MBs→b-DNACp-MBs; (2) Simultaneous hybridization of the target miRNA with the b-DNACp-MBs and labeling of the b-DNACp–miRNA heteroduplexes, immobilized onto the MBs with an anti-DNA–RNA hybrid antibody and ProtA–PolyHRP40 mixture solution pre-incubated for 1 h.	60
3	(1) b-DNACp immobilization onto Strep-MBs→b-DNACp-MBs; (2) Hybridization of the target miRNA with the b-DNACp-MBs; (3) Recognition of the b-DNACp–miRNA heteroduplexes immobilized onto the MBs by the anti DNA–RNA hybrid antibody and ProtA–PolyHRP40 mixture solution.	90
4	(1) b-DNACp immobilization onto Strep-MBs→b-DNACp-MBs; (2) Hybridization of the target miRNA with the b-DNACp-MBs; (3) Recognition of the b-DNACp–miRNA heteroduplexes immobilized onto the MBs by the anti DNA-RNA hybrid antibody; (4) Labeling of the anti DNA–RNA hybrid antibody with ProtA–PolyHRP40.	120

**Table 3:** Analytical characteristics obtained for the determination of miRNA-21 with the developed biosensor.

Parameter	Value
R *	0.9995
Slope, nA·pM^−1^	55.3 ± 0.9
Intercept, nA	228 ± 45
Linear range, pM	1.0–100
Limit of detection (LOD) **, pM	0.4
Limit of quantification (LQ) ***, pM	1.0

*: Pearson´s correlation coefficient in least squares regression analysis. **, ***: estimated according to the 3 × s_b_/m and 10 × s_b_/m criterion, respectively, with s_b_: standard deviation (*n* = 10) for measurements performed in the absence of miRNA-21, and m: slope value of the calibration plot shown in Figure 4.

**Table 4:** Sensitivity obtained for different working conditions checked in the assessment of assay time shortening.

b-DNACp-MBs-Mixture Solution Incubation Time, min	[Anti DNA–RNA Hybrid Antibody], µg·mL^−1^	[ProtA–HRP40], μg·mL^−1^	Slope, nA·nM^−^^1^	Sensitivity, %
30	2.0	2.0	55,214 ± 921	100
15	2.0	2.0	34,843 ± 2542	63.1
	4.0	2.0	30,844 ± 4494	55.9
	2.0	4.0	53,899 ± 659	97.6
	4.0	4.0	48,862 ± 4269	88.5

**Table 5:** Determination of the endogenous content of miRNA-21 (in amol per ng of RNA_t_) in human cells and breast tissues.

Sample		miRNA-21 (*n* = 3)	T/NT * Ratio	Contents Found by Other Authors
Cells	MCF-10A	0.79 ± 0.14	2.95	0.93 [35]
				1.02 [25]
	MCF-7	2.33 ± 0.54		3.3 [35]
				3.1 [25]
Breast tissues	NT1	0.94 ± 0.12	2.06–2.99	0.1−1.5 [35]
	NT2	0.80 ± 0.20		
	T1	2.81 ± 0.43		0.4−3.0 [35]
	T2	1.65 ± 0.20		

* miRNA-21 amount found in tumoral (T) vs non-tumoral (NT) tissues ratio.

**Table 6:** Oligonucleotides used in this work.

Oligonucleotide	Sequence (5′→3′)
b-antiDNA-21 Cp	5′-TCAACATCAGTCTGATAAGCTA-Biotin-3′
Target miRNA-21	5′-UAGCUUAUCAGACUGAUGUUGA-3′
1-central base mismatched miRNA-21 (miRNA-21 1-m(c))	5′-UAGCUUAUCAAACUGAUGUUGA-3′
1-terminal base mismatched miRNA-21 (miRNA-21 1-m(t))	5′-UAGCUUAUCAGACUGAUGUUGG-3′
miRNA-155	5′-UUAAUGCUAAUCGUGAUAGGGGU-3′
miRNA-223	5′-CGUGUAUUUGACAAGCUGAGUU-3′
